# Turnover in local parasite populations temporarily favors host outcrossing over self‐fertilization during experimental evolution

**DOI:** 10.1002/ece3.4150

**Published:** 2018-06-11

**Authors:** Zachary R. Lynch, McKenna J. Penley, Levi T. Morran

**Affiliations:** ^1^ Department of Biology Emory University Atlanta Georgia

**Keywords:** adaptation, experimental evolution, host mating system evolution, outcrossing, parasite turnover, self‐fertilization

## Abstract

The ubiquity of outcrossing in plants and animals is difficult to explain given its costs relative to self‐fertilization. Despite these costs, exposure to changing environmental conditions can temporarily favor outcrossing over selfing. Therefore, recurring episodes of environmental change are predicted to favor the maintenance of outcrossing. Studies of host–parasite coevolution have provided strong support for this hypothesis. However, it is unclear whether multiple exposures to novel parasite genotypes in the absence of coevolution are sufficient to favor outcrossing. Using the nematode *Caenorhabditis elegans* and the bacterial parasite *Serratia marcescens*, we studied host responses to parasite turnover. We passaged several replicates of a host population that was well‐adapted to the *S. marcescens* strain Sm2170 with either Sm2170 or one of three novel *S. marcescens* strains, each derived from Sm2170, for 18 generations. We found that hosts exposed to novel parasites maintained higher outcrossing rates than hosts exposed to Sm2170. Nonetheless, host outcrossing rates declined over time against all but the most virulent novel parasite strain. Hosts exposed to the most virulent novel strain exhibited increased outcrossing rates for approximately 12 generations, but did not maintain elevated levels of outcrossing throughout the experiment. Thus, parasite turnover can transiently increase host outcrossing. These results suggest that recurring episodes of parasite turnover have the potential to favor the maintenance of host outcrossing. However, such maintenance may require frequent exposure to novel virulent parasites, rapid rates of parasite turnover, and substantial host gene flow.

## 1 INTRODUCTION

One of the central mysteries in evolutionary biology is the overwhelming prevalence of sexual reproduction via outcrossing in plant and animal species. Compared to self‐fertilization, outcrossing entails substantial costs, including the twofold cost of males or the cost of meiosis (Lively & Lloyd, 1990; Maynard Smith, 1978; Williams, 1975). The ubiquity of outcrossing in the face of such costs suggests that outcrossing lineages enjoy significant benefits over evolutionary time, relative to selfing (Goldberg et al., 2010) or asexual lineages (Bell, 1982; Maynard Smith, 1978). The Red Queen hypothesis predicts that host–parasite coevolution can favor the long‐term maintenance of outcrossing (Bell, 1982; Hamilton, 1980; Jaenike, 1978). Parasites are predicted to incur selection to infect the most common host genotypes, which can impose negative frequency‐dependent selection on host populations (Koskella & Lively, 2009). Outcrossing hosts can produce genetically diverse offspring by exchanging genetic material across lineages and recombining beneficial mutations with different origins into novel or rare genotypes. Conversely, self‐fertilizing hosts are more likely to produce offspring with common genotypes that are predicted to suffer disproportionately from parasite infection. The fitness advantage that outcrossed offspring gain from reduced parasitism can fluctuate over time; if this advantage periodically outweighs the costs of sex, then outcrossing will be maintained in the long term (Vergara, Jokela, & Lively, 2014). Many studies of natural and experimental host–parasite coevolution have provided support for the Red Queen hypothesis (Dybdahl & Lively, 1998; Jokela, Dybdahl, & Lively, 2009; King, Delph, Jokela, & Lively, 2009; Koskella & Lively, 2009; Lively & Dybdahl, 2000; Masri et al., 2013; Morran, Schmidt, Gelarden, Parrish, & Lively, 2011; Slowinski et al., 2016; Vergara et al., 2014).

Although the Red Queen hypothesis specifically invokes negative frequency‐dependent selection driven by host–parasite coevolution as a mechanism that can favor the long‐term maintenance of outcrossing, other sources of recurring environmental change could have similar effects. If adaptive landscapes are frequently shifting over time, outcrossing lineages may gain substantial fitness advantages by producing genetically diverse offspring and assembling beneficial mutations from multiple lineages into novel genotypes. Theory predicts that natural selection will favor increased outcrossing and recombination if the sign of epistasis for fitness changes every two to five generations, such that genotypes with high fitness in any given generation become unfit a few generations later (Barton, 1995; Gandon & Otto, 2007; Peters & Lively, 1999). Conversely, populations evolving under relatively consistent environmental conditions will reach an adaptive peak, after which outcrossing is likely to break up adaptive gene complexes and epistatic relationships. This may lead to outbreeding depression and the re‐emergence of self‐fertilization or asexual reproduction (Lynch, 1991; Lynch & Deng, 1994). Therefore, it is critical to evaluate whether changing environmental conditions can produce sufficient shifts in adaptive landscapes to favor outcrossing and ultimately whether such environmental changes occur often enough to explain the maintenance of outcrossing in nature.

When spatial heterogeneity in selective pressures is coupled with frequent migration between environments, interactions between epistasis and selection will determine whether sex and recombination are favored. Sex and recombination can break down linkage between beneficial and deleterious alleles (Hill–Robertson interference) and promote the incorporation of incoming beneficial mutations into genotypes that confer high levels of fitness, but they may also break apart locally adapted genotypes (Hill & Robertson, 1966). These opposing effects may combine to favor sex and recombination if offspring from local × migrant crosses have higher average fitness than offspring from local × local and migrant × migrant crosses (Agrawal, 2009; Otto, 2009). Recent experimental evolution studies have demonstrated that sex can be favored when populations migrate between heterogeneous environments or adapt to new environments. Gray and Goddard (2012) passaged sexual and asexual yeast populations in two different selective environments with varying levels of migration between the environments. Only the sexual populations that experienced migration exhibited simultaneous adaptation to both environments. Becks and Agrawal (2010) used a facultatively sexual rotifer to study the effects of periodic migration between subpopulations in homogeneous and heterogeneous environments. They observed greater responsiveness to a sex‐inducing stimulus and higher frequencies of sexually derived offspring in heterogeneous environments. However, production of sexual offspring declined throughout the experiment in all treatments, suggesting that selective pressures in the experiment were insufficient to favor the long‐term maintenance of high levels of sex. Becks and Agrawal (2012) tracked rotifer populations adapting to new environments; immediately after the transitions, population densities declined while production of sexual offspring increased, and sexual offspring eventually exhibited higher fitness. However, as the transitioning populations reached new fitness plateaus, they began to resemble control populations, with stable population densities and fewer, less fit sexual offspring. Therefore, long‐term maintenance of high levels of sex and recombination may require frequently changing environmental conditions that impose strong selective pressures on local populations.

Experimental evolution studies using the nematode *Caenorhabditis elegans* have shown that outcrossing can be favored over selfing as populations respond to various selective pressures (Anderson, Morran, & Phillips, 2010). *Caenorhabditis elegans* populations consist of males and hermaphrodites. Hermaphrodites cannot mate with each other but can self‐fertilize or outcross with males (Brenner, 1974). Mutations at the mating system loci *xol‐1* and *fog‐2* can be exploited to generate obligately selfing and obligately outcrossing populations (Miller, Plenefisch, Casson, & Meyer, 1988; Schedl & Kimble, 1988). Morran, Parmenter, and Phillips (2009) exposed nematode populations to two different selection environments during 40‐ to 50‐generation evolution experiments: (1) a chemical mutagen coupled with a migration barrier and (2) the pathogenic bacterium *Serratia marcescens*. Obligately outcrossing populations showed stronger adaptation to the challenging environmental conditions than wild‐type and obligately selfing populations. Wild‐type populations evolved higher levels of outcrossing and exhibited stronger adaptation than obligately selfing populations. Morran et al. (2011) conducted a 30‐generation evolution experiment in which nematode populations were exposed to either a fixed strain of *S. marcescens* or a potentially coevolving *S. marcescens* population that was isolated from nematode carcasses every generation. Both parasite treatments led to significant increases in host outcrossing rates over the first eight generations, but only coevolving parasites selected for the maintenance of elevated outcrossing rates throughout the experiment. Slowinski et al. (2016) expanded on these results by showing that wild‐type *C. elegans* hermaphrodites (which are capable of self‐fertilization) rapidly invaded mutant obligate‐outcrossing populations when the mixed populations were exposed to avirulent or fixed parasite genotypes over 33 generations, but self‐fertilization did not invade *C. elegans* populations that were exposed to potentially coevolving parasites. Masri et al. (2013) did not find elevated host outcrossing rates during 48 generations of coevolution between *C. elegans* and the pathogenic bacterium *Bacillus thuringiensis*; nonetheless, their results still supported the Red Queen hypothesis. Although males were more susceptible to the parasite, outcrossing was maintained throughout the experiment and outcrossed offspring exhibited stronger resistance to the parasite.

Changes in the genetic composition of parasite populations may serve to generate environmental change for host populations, even when interactions are transient and coevolution is not possible. As a first step toward understanding the potential impact of recurring environmental change, we tested whether host outcrossing would be favored following single parasite turnover events. Specifically, is a change in the parasite genotype sufficient to favor outcrossing in host populations that have already adapted to a different genotype of the same parasite species? Starting with a *C. elegans* population that had previously adapted to a nonevolving *S. marcescens* population (strain Sm2170) during a 30‐generation evolution experiment (Morran et al., 2011; Penley, Ha, & Morran, 2017; Penley & Morran, 2017), we established an initial outcrossing rate of ~0.5 by manipulating the ratio of hermaphrodites to males. We made five replicates of this ancestral host population and passaged each of them with the *S. marcescens* strains CoSm, ES1, Rec320, and Sm2170 for 18 generations. The novel strains (CoSm, ES1, and Rec320) were derived from Sm2170 and experienced different selective pressures during previous evolution experiments: ES1 was selected to cause higher host mortality, Rec320 was selected to cause nonlethal infections, and CoSm was passaged apart from the host (Gibson et al., 2015; Morran et al., 2011). Therefore, we expected our parasite strains to have different initial levels of virulence, resulting in different host evolutionary trajectories and perhaps different outcrossing rates. We measured outcrossing rates every six generations to test whether higher levels of outcrossing would be favored as host populations adapted to novel parasite strains. At the end of our 18‐generation evolution experiment, we compared competitive fitness in the presence of parasites between ancestral hosts and evolved (generation 18) hosts to test whether novel parasite strains would induce faster rates of host adaptation. We also tested whether host resistance to the original parasite strain (Sm2170) would decrease as host populations adapted to the novel parasite strains, which would indicate cross‐resistance trade‐offs.

## 2 MATERIALS AND METHODS

### 2.1 Study system

*Caenorhabditis elegans* is a free‐living nematode that colonizes ephemeral bacterial blooms in rotting fruits and herbaceous stems. Conditions such as extreme temperatures, scarce food, and high population density cause *C. elegans* to enter a nonfeeding life stage known as dauer, in which larvae are resistant to environmental stresses and starvation. Dauer larvae actively seek invertebrate vectors for long‐distance dispersal to new bacterial blooms through nictation behavior, in which they stand on their tails and wave their heads (Cutter, 2015; Felix & Braendle, 2010; Frezal & Felix, 2015). *Caenorhabditis elegans* has an androdioecious mating system with males and self‐fertilizing hermaphrodites. The hermaphrodites cannot outcross with each other but may outcross with males (Brenner, 1974). All known natural strains predominantly self‐fertilize, although natural outcrossing rates are variable (Teotonio, Manoel, & Phillips, 2006). Given the characteristics of its mating system and its need for frequent migrations that may result in exposure to new parasites, *C. elegans* seems to be an appropriate model for studying how parasite turnover affects host outcrossing rates and adaptive potential. For our parasite, we used *S. marcescens*, a virulent bacterium that infects many plant and animal species (Grimont & Grimont, 1978). The *C. elegans*–*S. marcescens* interaction has been used to study the genetics of parasite infectivity, host resistance, and host avoidance behavior (Kurz et al., 2003; Mallo et al., 2002; Pradel et al., 2007; Schulenburg & Ewbank, 2004).

### 2.2 Host and parasite populations

*Caenorhabditis elegans* stock populations were maintained at 20°C in 10‐cm‐diameter Petri dishes filled with 30 ml of autoclaved nematode growth medium lite (NGM) (US Biological, Swampscott, MA, USA). These dishes were seeded with 200 μl of *Escherichia coli* strain OP50 culture that was grown overnight at 28°C in Luria‐Bertani broth (LB). After the *E. coli* lawns grew overnight at 28°C, the dishes were stored at 4°C for future use. *Caenorhabditis elegans* stock strains were derived from single wild‐caught individuals. We obtained the wild‐type strain CB4856 (from Hawaii, USA) and the GFP‐marked strain JK2735 from the Caenorhabditis Genetics Center (University of Minnesota, Minneapolis, MN, USA). Our ancestral *C. elegans* host population, EW2‐30, was derived from PX382, a systematically inbred variant of CB4856 (Morran et al., 2009). EW2‐30 resulted from a previous evolution experiment in which hosts were passaged with a nonevolving *S. marcescens* population (strain Sm2170) for 30 generations; the complete protocol is published in Morran et al. (2011). Briefly, a population of PX382 (the ancestral population of EW2‐30) was mutagenized with ethyl methanesulfonate and passaged on *Serratia* selection plates (SSPs), which required nematodes to migrate through live *S. marcescens* and ampicillin to reach their food source, *E. coli* strain OP50. Under these conditions, naïve host populations can suffer mortality rates up to 80% (Morran et al., 2009; Schulenburg & Ewbank, 2004). Only the offspring of nematodes that reached the food source proceeded to a new SSP to begin the next generation. At the end of the 30‐generation evolution experiment, EW2‐30 was frozen at −80°C for future use.

Our novel *S. marcescens* strains (ES1, Rec320, and CoSm) were derived from Sm2170 during previous evolution experiments. ES1 underwent selection for increased infectivity and virulence as it was passaged with a static CB4856 host population for 30 generations. Bacteria that killed nematodes after 24 hr of exposure were harvested every generation and used to infect the next generation of hosts; see Morran et al. (2011) for further details. Host and parasite populations can be copassaged to allow for coevolution; in this case, parasites are harvested from dead nematodes every generation and used to infect the offspring of surviving nematodes. Selection for reduced antagonism is also possible; a previous study identified hosts carrying mild upper intestine infections that were not cleared but did not cause death or prevent reproduction. During a 20‐generation evolution experiment, Gibson et al. (2015) copassaged offspring from infected parents with bacteria that caused those mild infections, resulting in the Rec320 parasite strain. As an alternative means of generating a less‐virulent parasite strain, CoSm was passaged for 20 generations on SSPs without nematodes; see Gibson et al. (2015) for further details regarding CoSm and Rec320.

### 2.3 Experimental evolution

Before starting our evolution experiment, we manipulated our ancestral host population (EW2‐30) to establish initial male frequencies of ~0.25 in each of the experimental populations. Groups of 20 L4 nematodes were transferred to NGM dishes seeded with OP50 and allowed to produce offspring; five dishes had a 1:1 ratio of hermaphrodites to males and five dishes had all hermaphrodites. Matings between hermaphrodites and males result in 50% male offspring, whereas selfing hermaphrodites produce ~0.02% male offspring, a frequency that approximates the rate of spontaneous X chromosome nondisjunction (Anderson et al., 2010). Therefore, mixing equal quantities of offspring from those 10 dishes resulted in a population with ~25% males. We created five replicates from this mix, transferring ~1,000 offspring to each replicate population, and passaged each of them for 18 generations on SSPs with four parasite strain treatments: CoSm, ES1, Rec320, and Sm2170. SSP construction and nematode transfers were performed using published protocols (Morran et al., 2011). Briefly, groups of ~1,000 L3–L4 nematodes were washed into M9 buffer and transferred to live *S. marcescens* lawns. Only the offspring of nematodes that successfully migrated through the parasite and a streak of ampicillin to reach their food source (*E. coli* strain OP50) were transferred to a new SSP to begin the next generation. Each of the five replicate ancestral populations was separately passaged with CoSm, ES1, Rec320, and Sm2170 for 18 generations, resulting in 20 evolved host populations. The replicate ancestral populations were frozen at −80°C at the beginning of the experiment and the experimentally evolved host populations were frozen at −80°C every six generations so they could be tested simultaneously in later assays.

### 2.4 Host mortality rate assays

Host mortality rates were assayed in 10‐cm‐diameter Petri dishes filled with 30 ml of NGM and seeded with 200 μl of *S. marcescens* culture that was grown overnight at 28°C in LB. The *S. marcescens* lawns grew overnight at 28°C, then groups of 200 L4 nematodes were transferred into the dishes. Dead nematodes were counted after 24 hr of parasite exposure, and 24‐hr host mortality rates were calculated as the number of dead nematodes divided by 200 transferred nematodes. *Serratia marcescens* strain Sm2170 is capable of establishing systemic infections and killing *C. elegans* within 24 hr (Morran et al., 2011), and host mortality is most accurately measured at this time point because the parasite disintegrates nematode carcasses after 24 hr. Before starting our evolution experiment, we used these assays to compare the virulence levels of our four *S. marcescens* strains (CoSm, ES1, Rec320, and Sm2170) toward the ancestral host population. For each of the five replicate ancestral host populations, we performed three technical replicates per treatment. The effects of parasite strain on 24‐hr mortality rates were analyzed using a generalized linear model (GLM) with quasi‐binomial error distribution and logit link function, and then, pairwise differences were assessed using Tukey's honest significant difference (HSD) tests. At the end of our evolution experiment, we used these assays to compare resistance to Sm2170 (to which our ancestral hosts were well‐adapted) across replicate host populations that had been passaged with each of the four parasite strains for 18 generations. Each experimental evolution treatment had five replicate host populations, and we performed two technical replicates per population. The effects of host evolution treatment on 24‐hr mortality rates were analyzed using a GLM with quasi‐binomial error distribution and logit link function. These analyses were performed in R version 3.2.3 (R Development Core Team, 2014) using the *multcomp* package (Hothorn, Bretz, & Westfall, 2008). ANOVA tables are provided in Appendix S1.

### 2.5 Measuring host outcrossing rates

Male frequencies were measured in each host population at the beginning of the experiment and after every six generations of experimental evolution. A transect of each experimental population was counted on the OP50 portion of the SSP every six generations. Approximately 200 L4 offspring were counted and sexed prior to passage to the next round of selection. The outcrossing rate for each host population was calculated by multiplying the male frequency by two after correcting for the number of males that typically result from spontaneous X chromosome nondisjunction (Stewart & Phillips, 2002). These data are presented in terms of outcrossing rates in Results but were analyzed in terms of male frequencies to enable the use of binomial GLMs. The effects of parasite strain, host generation, and their interaction on male frequencies in our host populations were analyzed using a GLM with quasi‐binomial error distribution and logit link function. We analyzed the effects of parasite strain on male frequencies at host generations 6, 12, and 18 using quasi‐binomial GLMs and then performed pairwise comparisons using Tukey's HSD tests. For each parasite strain treatment, we analyzed the effects of host generation on male frequencies using quasi‐binomial GLMs. We then performed pairwise comparisons using Tukey's HSD tests and tabulated the key differences: generation 6—ancestor, generation 12—ancestor, generation 18—ancestor, and generation 18—generation 12. These analyses were performed in R version 3.2.3 (R Development Core Team, 2014) using the *multcomp* package (Hothorn et al., 2008). ANOVA tables are provided in Appendix S1.

### 2.6 Male versus hermaphrodite susceptibility to parasites

We compared the survival rates of males and hermaphrodites from the ancestral host population when they were exposed to parasites within our selection regime. SSPs were prepared using published protocols (Morran et al., 2011), and groups of 222 hermaphrodites and 50 males were transferred into the *S. marcescens* side of the plates. Nematodes that migrated out of the *S. marcescens* side and were alive 48 hr after exposure to the parasite were counted as survivors. The ancestral host population was tested against each of the four parasite strains used for experimental evolution (CoSm, ES1, Rec320, and Sm2170) in five replicate plates per treatment. The effects of nematode sex on survival rate were assessed using GLMs with quasi‐binomial error distribution and logit link function. These analyses were performed in R version 3.2.3 (R Development Core Team, 2014). ANOVA tables are provided in Appendix S1.

### 2.7 Competitive fitness assays

We measured the competitive fitness of each ancestral and generation 18 host population relative to a common tester strain in our selective environment. In each assay, 100 nematodes from the focal population and 100 nematodes from the GFP‐marked strain JK2735 were transferred to the *S. marcescens* side of an SSP. Four days later, approximately 200 of the offspring on the OP50 side of the SSP were counted and assessed for GFP expression. The frequency of focal individuals in the offspring was then calculated as [1 − GFP frequency]; values above 0.5 indicate that the focal hosts out‐competed the tester strain. This measurement may underestimate the fitness of the focal hosts because any cross‐progeny of focal and tester individuals will express the dominant GFP marker (Morran, Parrish, Gelarden, Allen, & Lively, 2014; Morran et al., 2009). Host populations were frozen (−80°C) at the beginning of our evolution experiment and at generations 6, 12, and 18. For these experiments, we revived ancestral and generation 18 host populations and tested them simultaneously. This served two purposes: (1) to remove any effects of testing them at different times and (2) to reduce the likelihood that outcrossing activity would affect the frequency of GFP expression, because very few males survive freezing and thawing. For the ancestral hosts, we conducted three to five replicate assays with each parasite strain and calculated the mean frequency of focal offspring (FO_Ancestor_). For the generation 18 hosts, each replicate population was tested against the parasite strain it was passaged with and we performed two to four technical replicates per combination. We calculated percent changes in mean fitness by comparing the frequency of focal offspring from each generation 18 assay (FO_Gen18_) to the FO_Ancestor_ value corresponding to the same parasite strain: [(FO_Gen18_ − FO_Ancestor_) ÷ FO_Ancestor_]. These data violated the ANOVA assumptions of normality and homogeneity of variances. Therefore, we used a nonparametric Kruskal–Wallis *H* test to assess the effects of host evolution treatment (the parasite strain each host population was passaged with) on percent changes in mean fitness. Pairwise differences were assessed using Steel–Dwass tests. These analyses were performed in JMP 12.0 (SAS Institute, Cary, NC, USA).

## 3 RESULTS

**Figure 1:**
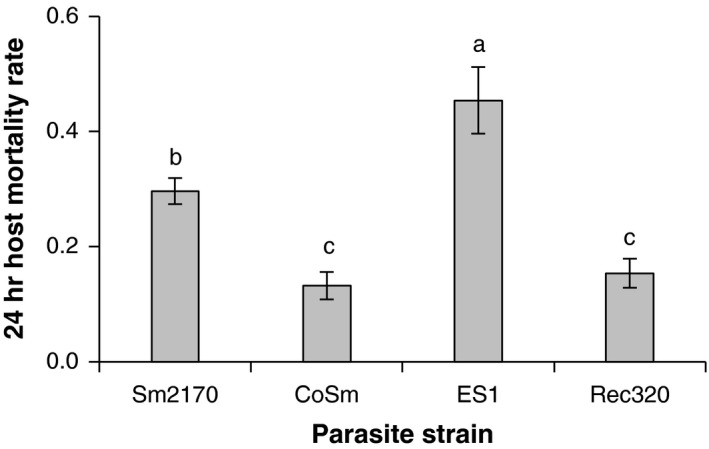
Mortality rates suffered by the ancestral *Caenorhabditis elegans* population after 24 hr of exposure to the four *Serratia marcescens* strains we used for experimental evolution (±1 *SEM*). Different letters indicate significant differences between parasite strains (Tukey's honest significant difference tests, *p *<* *.03). *N* = 5 replicate host populations with three technical replicates per treatment

Before starting our evolution experiment, we conducted host mortality rate assays to measure the virulence of our chosen *S. marcescens* strains toward the ancestral *C. elegans* population, which had previously adapted to Sm2170 during a 30‐generation evolution experiment (Morran et al., 2011; Penley et al., 2017). Our chosen parasite strains varied significantly in virulence toward the ancestral host population (Figure 1; *F*_3,16_ = 17.6, *p *<* *.0001); ES1 caused the greatest host mortality, Sm2170 caused intermediate host mortality, and CoSm and Rec320 caused the lowest host mortality (Tukey's HSD tests, *p *<* *.03). These results match our predictions based on the parasite strains' evolutionary histories: Sm2170 was the ancestor, ES1 was selected to cause higher host mortality, Rec320 was selected to cause nonlethal infections, and CoSm was passaged outside of the host (Gibson et al., 2015; Morran et al., 2011).

**Figure 2:**
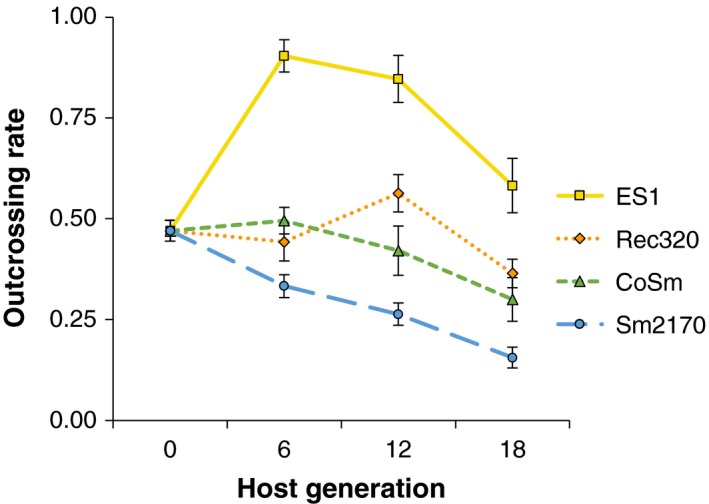
Changes in host outcrossing rates over time as *Caenorhabditis elegans* populations were passaged with four different *Serratia marcescens* strains for 18 generations (±1 *SEM*). *N* = 5 replicate host populations per treatment

Five replicates of the ancestral host population were passaged with each of the four parasite strains for 18 generations. We measured outcrossing rates in each host population every six generations to compare how they changed during adaptation to the novel strains (CoSm, ES1, and Rec320) versus a parasite strain to which the ancestral population was well‐adapted (Sm2170). There were significant effects of parasite strain (Figure 2; *F*_3,73_ = 60.4, *p *<* *.0001) and host generation (*F*_3,76_ = 15.9, *p *<* *.0001) on outcrossing rates. Changes in outcrossing rate over time differed across parasite strain treatments (parasite strain × host generation effect: *F*_9,64_ = 7.7, *p *<* *.0001). From generation 6 through the end of the experiment, outcrossing rates were highest in the ES1 treatment and lowest in the Sm2170 treatment, while the CoSm and Rec320 treatments maintained intermediate levels of outcrossing (Figure 2; Table 1). In host populations passaged with ES1, outcrossing rates were significantly higher than ancestral levels at generations 6 and 12, and then decreased back to ancestral levels by generation 18 (Figure 2; Table 2). In the CoSm and Rec320 treatments, host outcrossing rates were not significantly different between generation 0 and generation 6, 12, or 18. In the Sm2170 treatment, host outcrossing rates showed a marginally significant decline from generation 0 to generation 6 (*p* = .054) and were significantly lower at generations 12 and 18. Outcrossing rates decreased significantly between generations 12 and 18 in the ES1 and Rec320 treatments (Table 2), suggesting that any advantages of outcrossing over selfing in the context of novel parasites were only temporary. Overall, these results suggest that outcrossing was disadvantageous when hosts encountered parasites to which they were well‐adapted, but could be temporarily maintained or favored by selection in the presence of novel parasite genotypes, depending on their levels of virulence.

**Table 1:** Significance groups that resulted from calculating all pairwise differences in host outcrossing rates between parasite strain treatments (Figure 2) at generations 6, 12, and 18 (Tukey's honest significant difference tests, *p *<* *.015)

	ES1	Rec320	CoSm	Sm2170
Generation 6	A	BC	B	C
Generation 12	A	B	BC	C
Generation 18	A	B	BC	C

Group A: highest outcrossing; Group C: lowest outcrossing.

**Table 2:** The following contrasts in host outcrossing rates within each parasite strain treatment (Figure 2) are presented: generation 6—ancestor, generation 12—ancestor, generation 18—ancestor, and generation 18—generation 12. The sign of the test statistic (*t*_8_) indicates the direction of change in outcrossing rates over the time period in question (positive: increased outcrossing, negative: decreased outcrossing)

	Gen. 6–Gen. 0	Gen. 12–Gen. 0	Gen. 18–Gen. 0	Gen. 18–Gen. 12
ES1	*t*_8_ = 5.7 *p* = .0013	*t*_8_ = 5.0 *p* = .0029	*t*_8_ = 1.7 *p* = .32	*t*_8_ = −3.6 *p* = .022
Rec320	*t*_8_ = −0.49 *p* = .94	*t*_8_ = 1.5 *p* = .39	*t*_8_ = −2.0 *p* = .22	*t*_8_ = −3.6 *p* = .022
CoSm	*t*_8_ = 0.38 *p* = .97	*t*_8_ = −0.80 *p* = .81	*t*_8_ = −2.6 *p* = .087	*t*_8_ = −1.9 *p* = .26
Sm2170	*t*_8_ = −3.0 *p* = .054	*t*_8_ = −4.6 *p* = .0054	*t*_8_ = −7.4 *p *<* *.001	*t*_8_ = −3.1 *p* = .046

*p* values < .05 indicate statistically significant changes in outcrossing rates (Tukey's honest significant difference tests).

We calculated outcrossing rates in our host populations over time based on observed male frequencies. However, these measurements could be biased if males and hermaphrodites differ in susceptibility to any of the parasite strains used for experimental evolution. To address this possible concern, we compared the survival rates of males and hermaphrodites in the ancestral population within our selection regime. Nematodes that migrated out of the *S. marcescens* side of SSPs and were alive 48 hr after exposure to the parasite were counted as survivors. There was no significant difference in survival rate between males and hermaphrodites across the four parasite strains (Table 3; *F*_1,38_ = 1.41, *p* = .24). When the parasite strains were considered individually, male survival was significantly lower than hermaphrodite survival in the ES1 treatment (*F*_1,8_ = 6.52, *p* = .034), but no other significant differences were found (*F*_1,8_ < 2.2, *p *> .17). Therefore, the elevated outcrossing rates (and male frequencies) observed at generations 6 and 12 in the ES1 experimental evolution treatment (Figure 2; Table 2) occurred in spite of higher ancestral male susceptibility to this parasite strain (Table 3), suggesting that the adaptive benefits of outcrossing were sufficient to overcome this initial barrier.

**Table 3:** Mean survival rates of hermaphrodites and males in the ancestral *Caenorhabditis elegans* population within the selection regime (±1 *SEM*). Nematodes that migrated out of the *Serratia marcescens* side of *Serratia* selection plates and were alive 48 hr after exposure to the parasite were counted as survivors. Ancestral hosts were tested against each of the four parasite strains used for experimental evolution

	ES1	Rec320	CoSm	Sm2170
Hermaphrodite	0.359 ± 0.024	0.398 ± 0.015	0.134 ± 0.034	0.357 ± 0.032
Male	0.232 ± 0.037	0.404 ± 0.090	0.100 ± 0.027	0.264 ± 0.043

*N* = 5 replicate plates per treatment.

**Figure 3:**
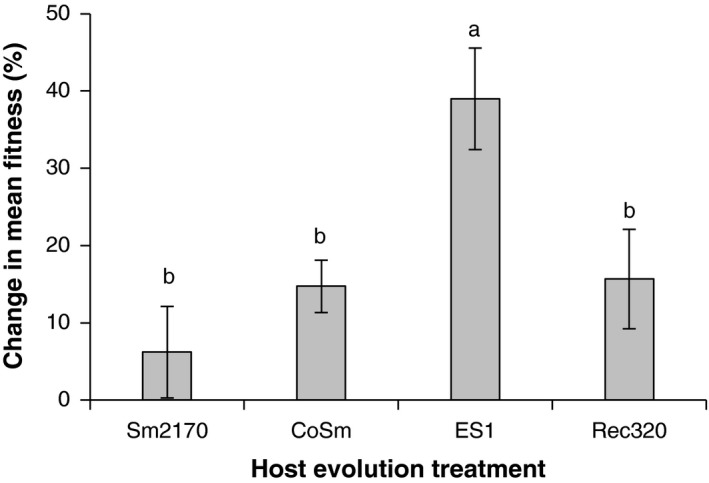
Percent changes in mean fitness for generation 18 hosts relative to their ancestors during exposure to the parasite strain each host was passaged with, as determined by competitive fitness assays against a GFP‐marked tester strain (±1 *SEM*). Different letters indicate significant differences between host evolution treatments (Steel–Dwass tests, *p *<* *.03). For ancestral hosts, *N* = 3–5 replicates per parasite strain; for generation 18 hosts, *N* = 5 replicate populations per treatment with two to four technical replicates per population

We evaluated the degree of host adaptation to each parasite strain over 18 generations of repeated exposure by assaying the competitive fitness of ancestral and generation 18 host populations. In each assay, equal numbers of nematodes from the focal host population and a GFP‐marked tester strain were mixed and exposed to the parasite strain the focal host was passaged with, using the same selective environment. After 4 days of exposure, we calculated the frequency of GFP‐marked offspring; frequencies below the starting level of 50% indicated that the focal hosts had greater competitive fitness. The percent change in mean fitness for generation 18 hosts relative to their ancestors differed significantly across host evolution treatments (Figure 3; χ^2^_3_ = 13.7, *p* = .003). Specifically, host populations that were passaged with ES1 exhibited the greatest rates of adaptation (Steel–Dwass tests, *p *<* *.03), but there were no significant pairwise differences between the CoSm, Rec320, and Sm2170 treatments (*p* > .8).

**Figure 4:**
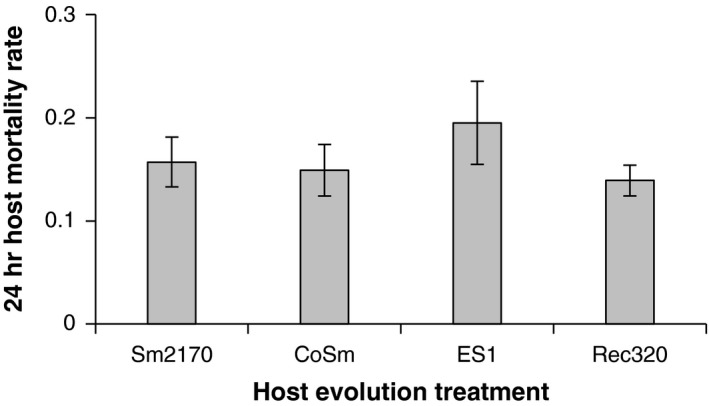
Mortality rates suffered by evolved *Caenorhabditis elegans* hosts after 24 hr of exposure to Sm2170 (±1 *SEM*). The host populations had been passaged with four different *Serratia marcescens* strains for 18 generations. *N* = 5 replicate host populations per evolution treatment with two technical replicates per population

We conducted host mortality rate assays to compare susceptibility to Sm2170 across our generation 18 host populations. Because the ancestral host population had previously adapted to Sm2170, decreased resistance against Sm2170 in host populations that were passaged with novel strains would suggest that resistance to different *S. marcescens* strains is subject to trade‐offs. However, there were no significant differences among generation 18 host populations in mortality rate following exposure to Sm2170 (Figure 4; *F*_3,16_ = 0.83, *p* = .50), suggesting a lack of cross‐resistance trade‐offs.

## 4 DISCUSSION

To study the effects of parasite turnover on host adaptation and outcrossing rates, we passaged replicate *C. elegans* populations that had previously adapted to the *S. marcescens* strain Sm2170 with three novel parasite strains and Sm2170 for 18 generations. Our novel strains were derived from Sm2170 during previous evolution experiments: ES1 was selected to cause higher host mortality, Rec320 was selected to cause nonlethal infections, and CoSm was passaged outside of the host (Gibson et al., 2015; Morran et al., 2011). Their levels of virulence toward the ancestral host population aligned well with their evolutionary histories; ES1 caused the greatest host mortality, Sm2170 caused intermediate host mortality, and CoSm and Rec320 caused the lowest host mortality (Figure 1). Changes in host outcrossing rates over time were influenced by both parasite novelty and parasite virulence. Host populations passaged with the most virulent novel parasite strain, ES1, exhibited the highest outcrossing rates throughout the experiment (Figure 2; Table 1) and their outcrossing rates increased significantly over the first 12 generations, an effect that was not observed in any other treatment (Table 2). ES1 was more virulent toward ancestral males than ancestral hermaphrodites within our selection regime (Table 3), suggesting that ancestral males surmounted an initial barrier before increasing in frequency in the ES1 evolution treatment. Conversely, in host populations passaged with Sm2170, outcrossing rates were lowest throughout the experiment (Table 1) and significantly below ancestral levels at generations 12 and 18 (Table 2). Host populations passaged with the less‐virulent novel strains, CoSm and Rec320, maintained their outcrossing rates at intermediate levels compared to those in the ES1 and Sm2170 treatments. These results suggest that selection may favor elevated host outcrossing rates during adaptation to highly virulent novel parasites. Furthermore, selection may favor the maintenance of host outcrossing during adaptation to novel parasite genotypes, even if they are less virulent than other parasite genotypes the host population recently encountered. However, the ES1 and Rec320 treatments showed significant declines in host outcrossing rates between generations 12 and 18 (Table 2), suggesting that single parasite turnover events can only temporarily favor the maintenance of host outcrossing.

Host populations passaged with the most virulent novel parasite strain, ES1, exhibited not only the highest outcrossing rates throughout the experiment (Figure 2), but also the strongest adaptation to parasites (Figure 3). However, links between parasite virulence, host outcrossing rates, and host adaptation were not clear across all treatments. Although host populations in the CoSm and Rec320 treatments generally maintained higher outcrossing rates than those in the Sm2170 treatment (Figure 2; Table 1), rates of host adaptation were not significantly different between these treatments (Figure 3). Furthermore, hosts did not lose resistance to Sm2170 as they adapted to the novel strains, suggesting that trade‐offs in resistance to different parasite strains did not occur (Figure 4). There are at least two possible explanations for this lack of cross‐resistance trade‐offs: (1) evolved resistance to the novel strains is independent of ancestral resistance to Sm2170 and (2) evolved resistance to the novel strains builds off of ancestral resistance to Sm2170. However, our experiments were not designed to test for mechanisms of resistance or virulence. These results may also be explained by the connected evolutionary histories of our host and parasite strains. Our ancestral hosts adapted to Sm2170 during a previous 30‐generation experiment (Morran et al., 2011; Penley et al., 2017). Our novel parasite strains were each derived from Sm2170; ES1 was selected for increased virulence over 30 generations (Morran et al., 2011), whereas CoSm and Rec320 were selected for lower virulence over 20 generations (Gibson et al., 2015). Perhaps parasite turnover events are more likely to produce the expected patterns of elevated host outcrossing rates, rapid host adaptation, and cross‐resistance trade‐offs when the parasites involved are more diverse and more virulent than those used in our study.

The patterns we observed in host outcrossing rates over time broadly agree with theoretical predictions and previous empirical results. Although outcrossing can increase the efficacy of selection during adaptation to changing environmental conditions (Agrawal, 2009; Otto, 2009), it is likely to be disfavored after a population reaches an adaptive peak because recombination and segregation will disassemble adaptive genotypes (Lynch, 1991; Lynch & Deng, 1994). Previous studies in which *C. elegans* populations were passaged with nonevolving *S. marcescens* found that outcrossing rates increased initially but decreased back to control levels by the end of the experiment; peak outcrossing rates were observed at generation 20 of 40 (Morran et al., 2009) and generation 8 of 30 (Morran et al., 2011). Furthermore, Slowinski et al. (2016) found that a wild‐type *C. elegans* lineage capable of self‐fertilization rapidly invaded mutant obligate‐outcrossing populations over 33 generations of exposure to both avirulent and fixed parasite genotypes, but could not invade host populations that were passaged with potentially coevolving parasites. Similar results have also been found in the absence of parasites; populations of facultatively sexual rotifers produced more sexual offspring during the initial stages of adaptation to new environments, but asexual offspring were favored after the populations reached new fitness plateaus (Becks & Agrawal, 2012). Therefore, it seems highly unlikely that single episodes of environmental change can favor the long‐term maintenance of high levels of outcrossing in species that normally self‐fertilize or reproduce asexually.

Host–parasite coevolution can generate the constantly shifting adaptive landscapes that are likely necessary for the long‐term maintenance of outcrossing, as shown in natural populations (Jokela et al., 2009; Vergara et al., 2014) and experimental systems (Masri et al., 2013; Morran et al., 2011). While other mechanisms may be capable of producing similar dynamics, it remains to be determined whether selective pressures apart from host–parasite coevolution can drive the long‐term maintenance of outcrossing. Here, we found short‐term adaptive increases in host outcrossing rates after the novel, and virulent parasite strain ES1 was introduced into replicate host populations. In the ancestral host population, males were more susceptible to ES1 than hermaphrodites (Table 3), yet males overcame this initial disadvantage to ultimately increase in frequency (Figure 2) and help the evolved populations achieve higher competitive fitness (Figure 3). Our results contrast slightly with those of Masri et al. (2013), who found that lower yet stable male frequencies were maintained during coevolution between *C. elegans* and a different bacterial parasite, *B. thuringiensis*. Male hosts were less resistant to the parasite and exhibited decreased sexual activity and increased escape behavior during exposure to the parasite. However, Masri et al. (2013) demonstrated a short‐term advantage of outcrossing that supports the Red Queen hypothesis: Offspring that resulted from outcrossing were more resistant to the parasite. Taken together, these results suggest that parasite‐mediated selection pressures have the potential to support the maintenance of host outcrossing. However, the evolutionarily optimal level of outcrossing may be higher or lower in different host–parasite interactions depending on the (potentially opposing) effects of parasites on male viability and effects of outcrossing on offspring resistance. When interactions are transient and coevolution is not possible, maintenance of host outcrossing may require frequent exposure to novel virulent parasites and rapid rates of parasite turnover to generate sufficient shifts in the adaptive landscape (Barton, 1995; Gandon & Otto, 2007; Peters & Lively, 1999).

One potential limitation to the long‐term maintenance of host outcrossing by frequent parasite turnover is that persistent directional selection imposed by parasites will likely deplete additive genetic variation in the host population. In an experimental evolution study comparing the responses of inbred and genetically variable *C. elegans* populations to the bacterial parasite *S. marcescens*, Parrish, Penley, and Morran (2016) found that inbred populations had reduced outcrossing rates and did not exhibit increased fitness, whereas genetically variable populations showed the opposite results. It is also important to consider how standing genetic variation can affect outcrossing rate dynamics in the absence of parasites. Teotonio, Carvalho, Manoel, Roque, and Chelo (2012) created high‐ and low‐diversity replicates of a hybrid *C. elegans* population derived from several wild isolates and passaged them for 100 generations in a novel laboratory environment. Male frequency was stably maintained around 20% in high‐diversity populations, and male frequency increased from 0% to near 14% in low‐diversity populations. Stewart and Phillips (2002) established male‐enriched populations of the *C. elegans* laboratory strain N2 and found that average male frequencies rapidly decreased from 45% to 7% over just 15 generations in benign laboratory conditions. Although the inbred populations in Teotonio et al. (2012) and the N2 populations in Stewart and Phillips (2002) can both be described as “low‐diversity” (Sivasundar & Hey, 2003), their origins and observed outcrossing rate dynamics were very different. Taken together, these results suggest that both the level and the nature of standing genetic variation in a population will affect outcrossing rate dynamics, depending on whether a population is experiencing inbreeding depression or outbreeding depression. The ancestral host population used in our study was chemically mutagenized before being passaged with the *S. marcescens* strain Sm2170 for 30 generations (Morran et al., 2011). Our results indicate that the ancestral host population possessed sufficient additive genetic variation to respond to selection pressure from a novel, virulent parasite over 18 generations and that high levels of outcrossing facilitated this response (Figures 2 and 3). However, for host outcrossing to be maintained in the long term, recurring parasite turnover may need to be coupled with periodic replenishment of additive genetic variation in the host population.

Host migration is a potential mechanism for mitigating the loss of additive genetic variation in host populations as they adapt to environmental changes, such as parasite turnover. Migration between different host populations can introduce novel genetic variation. Further, theory predicts that outcrossing may be favored in the context of frequent migration because sex can break down unfavorable genetic associations that are introduced from outside populations (Agrawal, 2009; Lenormand & Otto, 2000). An experimental evolution study in which sexual and asexual yeast populations were subjected to varying levels of migration between two different selective environments showed that only the sexual populations that experienced migration adapted to both environments (Gray & Goddard, 2012). Therefore, frequent host migration coupled with frequent environmental change may provide the most likely context for the maintenance of outcrossing, apart from host–parasite coevolution. Our results suggest that environmental change resulting from parasite turnover can favor host outcrossing in the short term, but further work is required to determine whether recurring episodes of host migration and parasite turnover can contribute to the long‐term maintenance of outcrossing. The natural context for interactions between these mechanisms also requires further investigation, because any explanation for the maintenance of outcrossing must ultimately account for the distribution of outcrossing in nature.

Host–parasite coevolution remains the most well‐supported mechanism for generating constantly shifting adaptive landscapes that may favor outcrossing over selfing despite the inherent costs of sex (Lively & Morran, 2014). However, our results suggest that periodic parasite turnover events could have similar effects, if only temporarily, in the absence of host–parasite coevolution. A significant challenge for future studies is to determine whether selection imposed by coevolving parasites is uniquely able to maintain host outcrossing or whether coevolving parasites represent one of multiple forms of parasite turnover that can favor the long‐term maintenance of host outcrossing.

## CONFLICT OF INTEREST

None declared.

## AUTHOR CONTRIBUTIONS

Conceptualization, Methodology, and Experimentation: Z.R.L., M.J.P., and L.T.M. Statistical Analysis: Z.R.L. and L.T.M. Writing (original draft): Z.R.L. and L.T.M. Writing (review and editing): Z.R.L., M.J.P., and L.T.M.

## DATA ACCESSIBILITY

All data from this manuscript are available at Dryad https://doi.org/10.5061/dryad.rf47cp2.
