# Supplementary material for: Turnover in local parasite populations temporarily favors host outcrossing over self‐fertilization during experimental evolution
Source: Ecol Evol. 2018 Jun 11;8(13):6652–62. doi: 10.1002/ece3.4150 (PMC6053587; doi:10.1002/ece3.4150)
Supplement: Supplementary file 1 [file ECE3-8-6652-s001.docx]

**SUPPORTING INFORMATION S1**

**Turnover in local parasite populations temporarily favors host outcrossing over self-fertilization during experimental evolution**

Zachary R. Lynch, McKenna J. Penley, and Levi T. Morran

ANOVA tables corresponding to each figure/table:

**Fig. 1**

| **Model Term** | **D.F.** | **Deviance** | **Residual D.F.** | **Residual Deviance** | **F** | **P-value** |
| --- | --- | --- | --- | --- | --- | --- |
| NULL |  |  | 19 | 445.98 |  |  |
| Parasite Strain | 3 | 342.15 | 16 | 103.83 | 17.59 | 2.56e-05 |

**Fig. 2**

| **Model Term** | **D.F.** | **Deviance** | **Residual D.F.** | **Residual Deviance** | **F** | **P-value** |
| --- | --- | --- | --- | --- | --- | --- |
| NULL |  |  | 79 | 969.04 |  |  |
| Generation | 3 | 128.19 | 76 | 840.85 | 15.93 | 7.64e-08 |
| Treatment | 3 | 485.94 | 73 | 354.92 | 60.39 | 2.20e-16 |
| Generation x  Treatment | 9 | 185.82 | 64 | 169.10 | 7.70 | 1.44e-07 |

**Table 3**

| **Model Term** | **D.F.** | **Deviance** | **Residual D.F.** | **Residual Deviance** | **F** | **P-value** |
| --- | --- | --- | --- | --- | --- | --- |
| NULL |  |  | 39 | 474.34 |  |  |
| Nematode Sex | 1 | 15.31 | 38 | 459.03 | 1.41 | 0.24 |

Analyzing the ES1 data alone from Table 3 (this was the only parasite strain to show a significant difference in susceptibility between males and hermaphrodites):

| **Model Term** | **D.F.** | **Deviance** | **Residual D.F.** | **Residual Deviance** | **F** | **P-value** |
| --- | --- | --- | --- | --- | --- | --- |
| NULL |  |  | 9 | 35.26 |  |  |
| Nematode Sex | 1 | 15.41 | 8 | 19.84 | 6.52 | 0.034 |

**Fig. 4**

| **Model Term** | **D.F.** | **Deviance** | **Residual D.F.** | **Residual Deviance** | **F** | **P-value** |
| --- | --- | --- | --- | --- | --- | --- |
| NULL |  |  | 19 | 96.21 |  |  |
| Host Evolution Treatment | 3 | 13.17 | 16 | 83.03 | 0.83 | 0.50 |
